# Multivariate Statistical Analysis for Mutual Dependence Assessment of Selected Polyphenols, Organic Acids and Metals in Cool-Climate Wines

**DOI:** 10.3390/molecules27196566

**Published:** 2022-10-04

**Authors:** Magdalena Fabjanowicz, Vasil Simeonov, Marcin Frankowski, Wojciech Wojnowski, Justyna Płotka-Wasylka

**Affiliations:** 1 Department of Analytical Chemistry, Faculty of Chemistry, Gdańsk University of Technology (GUT), 11/12 Narutowicza Street, 80-233 Gdańsk, Poland; 2 Faculty of Chemistry and Pharmacy, University of Sofia, 1 James Bourchier Blvd., 1126 Sofia, Bulgaria; 3 Deparment of Analytical and Environmental Chemistry, Faculty of Chemistry, Adam Mickiewicz University, Uniwersytetu Poznańskiego 8, 61-614 Poznań, Poland; 4 Department of Chemistry, University of Oslo, P.O. Box 1033-Blindern, 0315 Oslo, Norway; 5 Department of Analytical Chemistry, Faculty of Chemistry and BioTechMed Center, Gdańsk University of Technology (GUT), 11/12 Narutowicza Street, 80-233 Gdańsk, Poland

**Keywords:** ICP-MS, chemometric analysis, cool-climate wines, resveratrol, multivariate statistics, chemical compound

## Abstract

Polyphenols, organic acids and metal ions are an important group of compounds that affect the human health and quality of food and beverage products, including wines. It is known that a specific correlation between these groups exist. While wines coming from the New World and the Old World countries are extensively studied, wines coming from cool-climate countries are rarely discussed in the literature. One of the goals of this study was to determine the elemental composition of the wine samples, which later on, together as polyphenols and organic acids content, was used as input data for chemometric analysis. The multivariate statistical approach was applied in order to find specific correlations between the selected group of compounds in the cool-climate wines and the features that distinguish the most and differ between red and white wines and rosé wines. Moreover, special attention was paid to resveratrol and its correlation with selected wine constituents.

## 1. Introduction

Wine is among the most often consumed alcoholic drinks around the world, mainly due to its sensory traits and health benefits [1]. Wine contains many compounds from different groups such as alcohols, acids, saccharides, minerals and others including biogenic amines, phenolic compounds and organic acids [2]. All of these compound groups have been shown to be, to a varying degree, related to wine quality as well as its health-promoting properties [3,4]. Polyphenols are among the group of compounds which not only to a high degree determine the organoleptic properties of wine such as flavor, taste and color, but also health benefits related to its consumption due to their antioxidant and cardioprotective properties [3]. In fact, the relation between phenolic composition and the commercial value of wine has been reported [5,6]. This is why the knowledge on the phenolic composition of wine is of high importance. 

Another group of compounds that also has a significant impact on the quality of wine are organic acids. These compounds are usually considered to have a weak antioxidant power and thus are often discarded in the extraction process, which is why their pharmacological effects have not been sufficiently studied [7]. However, an increasing amount of research focused on this group of compounds is performed nowadays, with special emphasis on the positive effects of organic acids on the human body, such as their antibacterial and anti-inflammatory activity and promotion of the absorption of specific elements [8,9]. In addition, organic acids play a principal role in maintaining the quality and nutritional value of food. The same role can also be assigned to some metals present in the wine. On the other hand, the content of trace metals in wine has to be controlled due to their adverse effect on human health and wine quality [10]. 

It is well-known that the occurrence of some of the aforementioned groups of compounds is interrelated—information that is valuable to manufacturers and consumers alike, mostly due to the possible adverse effect of ingestion of, e.g., heavy metals on the human body. Several interactions between phenolic compounds and metal content in wine have been reported. Bai et al. (2004) indicated that all the transition metals can form complexes with a particular flavanol (rutin) [11]. Esparza et al. (2004) studied the Zn and Cu interactions with three selected flavonoids (catechin, quercetin and rutin) [12]. In the case of the comparison of the total concentration of metals, polyphenols and anthocyanins, the concentrations of the latter increased, while the content of Fe and Cu decreased, giving rise to an inverse relationship. This can be explained by the participation of these metals in condensation reactions of tannins and anthocyanins. 

To the best of our knowledge, there are no studies on the correlations between the content of specific phenolic compounds such as resveratrol, organic acids and metals in wines produced from grapes cultivated in cool climates. Based on a literature review, it can be summarized that interactions between several parameters exist and are proven; therefore, we believe that interactions between the mentioned group of compounds may also exist. Hence, the aim of this work was to determine the elemental composition of the studied wine samples and, together with the results of the polyphenols and organic acids content, evaluate the parameters that characterizes the wine originating from cool climates. Emphasis was put on the differentiation between white, rosé and red wines. Moreover, the work aimed to determine specific correlations between chemical compounds determined in different kinds of wines produced in a cool climate. Particular attention was paid to resveratrol and its correlation with other compounds.

## 2. Results

The elemental analysis included the determination of Ag, Al, As, B, Ba, Be, Ca, Cd, Co, Cr, Cu, Fe, Hg, K, Li, Mg Mn, Na, Ni, Pb, Sb, Se, Sn, Sr, Ti, Tl, V, Zn and Zr, and was performed based on the method previously published by [13]. Results of the determination of these elements are gathered in Table S2, while the concentrations of organic acids and polyphenols are listed in Table S3. 

The gathered data set consists of 23 objects (wine samples from different regions and with different quality parameters) described by 43 chemical descriptors (variables). One of the descriptors (coded as FeA, or ferulic acid) is excluded from the chemometric analysis since it lacks any level of variation (all samples have one and the same concentration level recorded).

The major goals of the multivariate data mining include: identification of groups of similarity (patterns, clusters) between the objects of the study (wine samples) or between the descriptor variables; determination of latent factors responsible for the data structure and of the descriptors responsible for the formation of the similarity patterns of objects. In this way, it becomes possible to interpret the analytical data in a specific way, namely, reaching a classification of the different samples into several classes of analogy and explaining the reasons for achieving this partitioning of the samples. It makes it possible to better interpret the wine quality of each identified class and to select specific descriptors responsible for different wine quality classes.

In order to fulfil the goals of the study, several established multivariate statistical methods for intelligent data analysis are used—hierarchical and non-hierarchical (K-means) clustering and principal components analysis. All the mentioned methods are fully described in [14] and do not need detailed description.

**Figure 1:**
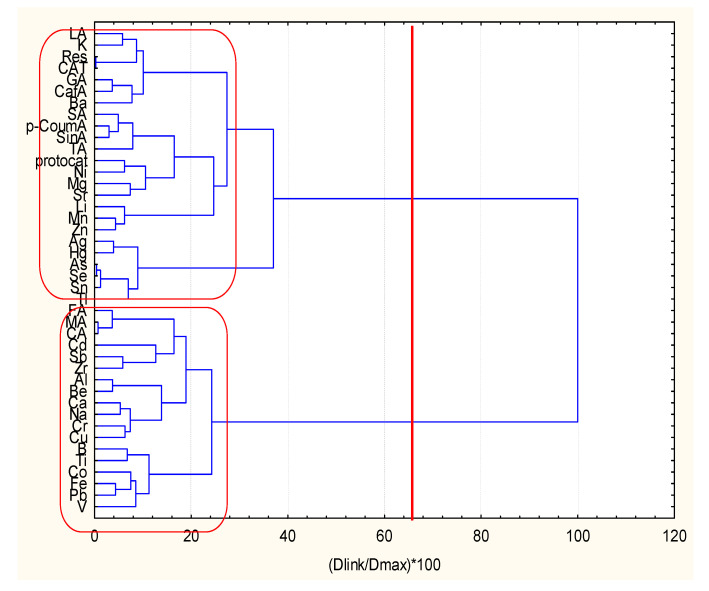
Hierarchical dendrogram for clustering of 42 variables.

In Figure 1 the hierarchical clustering of 42 variables is presented. HCA was performed after the z-standardization of the input raw data, squared Euclidean distances as similarity measure, Ward’s method of linkage and Sneath’s test for cluster significance. 

Two major clusters were identified. The members (18) in cluster 1 (the lower part of the dendrogram) are FA, MA, CA, Cd, Sb, Zr, Al, Be, Ca, Na, Cr, Cu, B, Ti, Co, Fe, Pb and V, and the remaining 24 variables belong to cluster 2 (the upper part of the dendrogram) as follows ( LA, K, Res, CAT, GA, CafA, Ba, SA, P-CoumA, SinA, TA, protocat, Ni, Mg, Sr, Li, Mn, Zn, Ag, Hg, As, Se, Sn, Tl). It is important to note that cluster 1 includes dominantly metal descriptors (15 out of a total of 18 members) and only 3 organic acid descriptors, and it could be conditionally named “soil major metal descriptors components”. The second cluster consists of 12 organic acids descriptors and 12 metal descriptors. Its content reveals the organic soil composition and soil trace metal descriptors (Ag, Hg, As, Se, Sn, Tl, Ni, Sr belonging to the group of potentially toxic elements), which form a subcluster in cluster 2 and the rest of some major soil metal components (Mg, Li, Mn, Zn). Therefore, cluster 2 could be conditionally marked as “organic descriptors components and soil trace metal components”.

It can be concluded that two specific patterns of descriptors determine the quality of the wine samples of interest. In Figure S1 the hierarchical dendrogram for clustering of the 23 wine samples is presented. Again, two patterns of similarity could be easily distinguished—the lower cluster 1 includes samples 11–23, and the upper cluster 2-samples 1–10. Both groups are well-separated and represent two different types of wine quality. This separation is reasonable if the input data are carefully checked. The wine samples from 1 to 10 are red wines, dominantly from the grape types Rondo and Regent, and dry with respect to sugar content, since the other 13 (11–23) are white and rosé wines originating from various grape types, and semidry and sweet according to the sugar content.

In order to better understand this sample partitioning, K-means clustering was applied as an unsupervised pattern recognition technique. The priori hypothesis required separation of both samples and variables into two clusters. The results of the non-hierarchical clustering confirmed entirely those of the hierarchical separation (white, rosé and red wine classes). Additionally, a plot was constructed (Figure S2), which presents the averages of each variable for each one of the identified clusters. This plot makes it possible to determine the specific descriptors responsible for the chemical-caused partitioning of the wine samples.

Cluster 1 includes samples (11–23) and cluster 2 those from 1 to 10. It has to be mentioned that sample 1 differs significantly from the other members of cluster 2 and could be defined as an outlier. Not all of the variables on the plot are presented, but they always follow the sequence used in the input table (Table S4) from LA (variable 1) to Zr (variable 42), with a distance of 3 not indicated on the axis members.

The results of the partitioning procedure indicated that samples 1–10 (cluster 2) are characterized (with respect to the organic acid components) by high levels of LA, protocat, p-CoumA, GA, CafA, SinA, Res and CAT, and by lower levels of FA, MA and CA as compared to the members of cluster 1; the organic acids SA and TA are almost of one and the same level for the members of both clusters. If the metal descriptors are compared, samples 1–10 are characterized by higher levels of Ag, As, Be, Hg, K, Mg, Sn, Se, Sr and Ti, and lower levels for Al, Ba, Ca, Cd, Co, Cr, Cu, Fe, Li, Na, Pb, V and Zr, as compared to the concentration levels of the metal components in cluster 1. For the rest of the metal species B, Mn, Ni, Sb, Zn and Tl, the concentration levels for both clusters are almost equal. The first stage of variables (descriptors) reduction proves that SA, TA, B, Mn, Ni, Sb, Zn and Tl could be eliminated as sufficient parameters for the determination of the wine quality. A better partitioning of both clusters with less variables (34 instead of 42) is indicated in Figure S3.

This multivariate statistical method determines the data set structure and reveals hidden factors (principal components) responsible for this structure. The definition of the new directions in the space of the variables (latent components) leads to the reduction of the initial number of variables and options to present the data location on a plane. Very often this approach is called projection method. Our goal when applying factor analysis is to try to reduce the variable numbers and to select specific descriptors able to explain the data structure.

The result of the analysis is a table of the so-called factor loadings, which interpret the relationship between the input variables within the newly defined latent components. Additionally, it is possible to use another table of the so-called factor scores representing the new space coordinates of the objects and indicating patterns of similarity (classes) between the objects. In Table S5 the factor loadings for all 42 variables are listed. 

**Figure 2:**
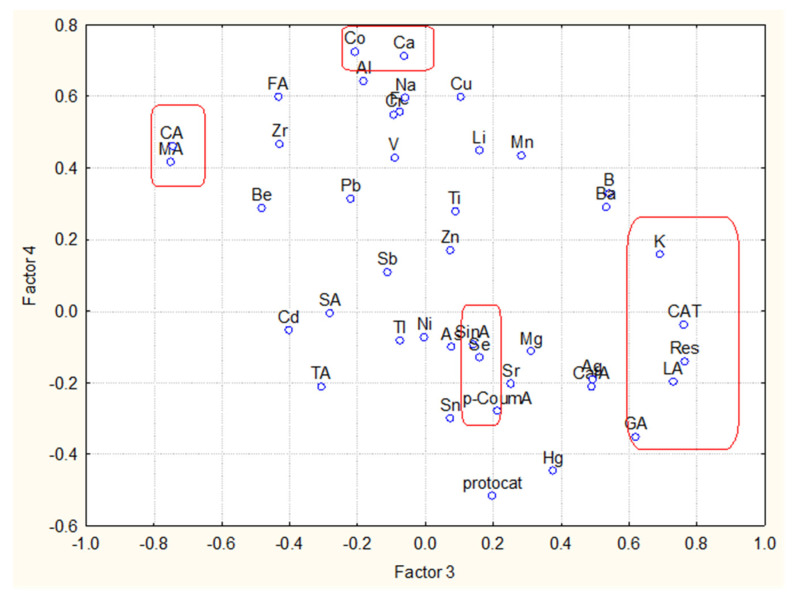
Biplot of factor loadings for factor 3 vs. factor 4.

**Figure 3:**
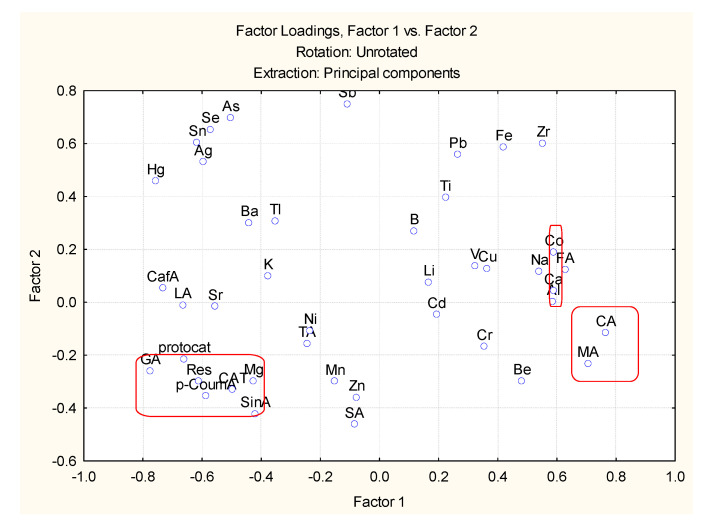
Biplot of factor loadings for factor 1 vs. factor 2.

The loadings marked by bold are statistically significant and of substantial interest for the data interpretation. Four latent factors explain over 80% of the total variance. The numbers of each factor are related to the percentage of variance explanation by each one of them. Keeping in mind the factor loadings values, we could select significant descriptors from the list of all variables (the higher the factor loading, the higher the significance of the variable). In Figure 2 and Figure 3 the biplot of the factor loadings for two combinations of latent factors are shown.

The variables with high factor loadings with respect to factor 3 and factor 4 are K, CAT, LA, Res, GA, CA and MA. The other two marked groups, Co and Ca, and SinA and p-CoumA, do not indicate high loadings to factors 3 and 4, but as seen in Figure 3 they show high loadings with respect to factors 1 and 2. 

**Figure 4:**
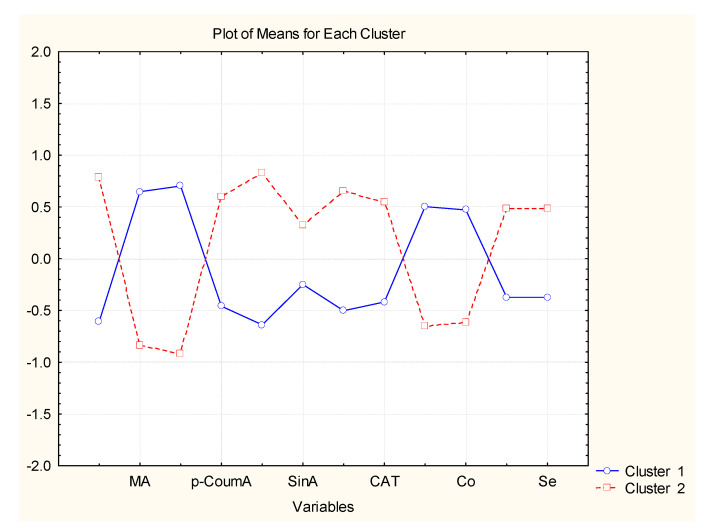
Plot of mean values (standardized) for each selected descriptor (final reduced number) for each identified cluster.

From all the available variables, one could select 12 as possible descriptors for the easy and reliable partitioning of the 23 wine objects: LA, MA, CA, p-CoumA, GA, SinA, Res, CAT, Ca, Co, K and Se. Most of the chosen descriptors are organic compounds, but there are also 4 metal descriptors obviously related to the soil specificity. When using only these 12 descriptors, one could reach a very good partitioning between the objects (Figure 4). Two groups (classes) are well-separated by the difference of the concentration levels of the 12 descriptors. Again, one of the clusters formed using only 12 variables is the class of red wine (cluster 2) and the other one—that of white wines (cluster 1).

**Figure 5:**
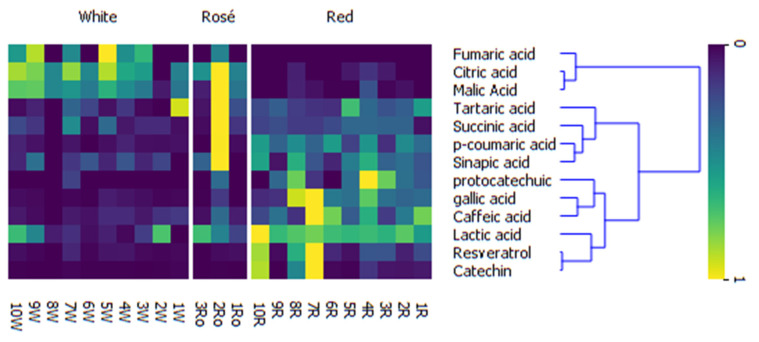
Heat map combined with hierarchical clustering with averaged linkage showing the difference in concentration of selected organic compounds and polyphenols in samples of white, red and rosé wines.

As compared to the separation shown in Figures S2 and S3, the presented partitioning is much better and simpler. The concentration of resveratrol—which is of particular importance due to health-beneficial properties including anti-obesity, anti-aging, anti-cancer and lipid/glucose metabolism-regulating properties, as well as reducing oxidative damage and inflammation, neuroprotection and chemoprevention [15,16]—varies significantly between the samples of white, red and rosé wines, and also within the red wines (Figure 5), with the highest concentration in the 7R and 10R samples. Based on the ReliefF scoring (Table S6), its concentration is best predicted based on the concentration of (in decreasing order) Zr, K, B, Pb and Ba; however, in the case of Zr and Pb, the correlation between their concentration in wine samples is negative.

## 3. Materials and Methods

### 3.1. Reagents, Chemicals and Standards

The ICP IV multi-element standard (Merck KGaA, Darmstadt, Germany) and single standards—As, Sb, Se, Mo and V (Merck KGaA, Darmstadt, Germany), Hg (Merck KGaA, Darmstadt, Germany)—were used for the calibration of ICP-MS. Sc, Rh, Tb and Ge in supra-pure 1% HNO3 (Merck KGaA, Darmstadt, Germany) were used as internal standards, and deionized water obtained from the Milli-Q Direct 8 Water Purification System (Merck-Millipore, Molsheim, France) was used for sample dilution. 

### 3.2. Samples

For the analysis, samples drawn from 23 bottles of wine originating from different regions of Poland were used (10 red wines, 10 white wines and 3 rosé wines). All samples were stored at room temperature (21 °C) and were protected from light. Details regarding the analyzed wines are gathered in Table S1.

### 3.3. Instrumentation

The elemental analysis with the use of ICP-MS was performed on the ICP-MS 2030 (Shimadzu, Kyoto, Japan) with the operation conditions summarized in Table 1.

### 3.4. Chemometric Analysis

The software package used for multivariate data cluster analysis (hierarchical and non-hierarchical clustering) was STATISTICA 8.0 (New York, NY, USA). Standardization of raw input data, application of squared Euclidean distances as similarity measures, Ward’s method of linking, Sneath’s test for cluster significance and a hierarchical dendrogram as a graphical output were all necessary to carry out the hierarchical clustering operation. In addition, non-hierarchical clustering of items using K-means was conducted. This is a typical supervised pattern recognition method, in which objects or variables are sorted into an a priori predetermined number of clusters; this number should prove or disprove preliminary hypotheses proposed by experts or particular preliminary data. In addition, the Varimax rotation mode was used for factor analysis. LOD/2 values were used to fill in for missing data. 

The results of elemental analysis and the chromatographic results of polyphenols and organic acids content [17] were used as inputs for further supervised analysis, which was carried out using Orange v. 3.28 and Scikit-Learn v. 0.23 Python packages [18,19]. The relative values of variables with respect to grape type were visualized using heat maps combined with hierarchical clustering with average linkage. In the particular case of resveratrol, a supervised method (ReliefF) was used to rank the importance of features obtained using elemental analysis [20].

## 4. Conclusions

The study was focused on the cool-climate wines whose chemical characteristic is still not well-known. Due to this fact, this work raised an important issue of specific correlation between selected chemical compounds present in examined wine. Application of chemometric analysis with a multivariate statistical approach (which could be considered as a training procedure) could help in the categorization of wines with unknown origin to some of the classes above using rapid analytical testing. It is readily seen that the most crucial are the concentration of LA, p-CoumA, GA, SinA, Res, CAT, K and Se, which are higher in red wines than for the same descriptors for white and rosé wines. Conversely, the white wines and rosé wines have higher concentrations of MA, CA, Ca and Co. Furthermore, ReliefFscoring indicates a possible correlation between resveratrol and several metals such as Zr, K B, Pb and Ba, which needs further investigation by the application other analytical instruments.

## Figures and Tables

**Table 1:** ICP-MS measurement conditions.

Parameters	ICP-MS
Radio frequency power generator [kW]	1.2
Gas type	Argon
Plasma gas flow rate [L min^−1^]	8.0
Auxiliary gas flow rate [L min^−1^]	1.1
Nebulization gas flow rate [L min^−1^]	0.7
Torch	Mini-torch (quartz)
Nebulizer	Coaxial
Spray chamber temperature [°C]	3
Drain	Gravity fed
Internal standard	Automatic addition
Sampling depth [mm]	5
Collision cell gas flow (He) [mL min^−1^]	6.0
Cell voltage [V]	−21
Energy filter [V]	7.0
Number of replicates	3
Integration conditions/number of scans	10

## Data Availability

Not applicable.

## Supplementary Materials

The following supporting information can be downloaded at https://www.mdpi.com/article/10.3390/molecules27196566/s1. Figure S1: hierarchical dendrogram for clustering of 23 wine samples (objects); Figure S2: plot of mean values (standardized) for each variable for each identified cluster; Figure S3: plot of mean values (standardized) for each variable (reduced number) for each identified cluster; Table S1: wine sample characteristics; Table S2: elemental composition of studied wine samples [µg/mL] and * [mg/mL]; Table S3: organic acids and polyphenols concentration in studies’ wine samples [mg/mL]; Table S4: input table; Table S5: factor loadings; Table S6: ReliefFscoring of chemical elements with relation to the concentration of resveratrol.
