# Supplementary material for: Multivariate Statistical Analysis for Mutual Dependence Assessment of Selected Polyphenols, Organic Acids and Metals in Cool-Climate Wines
Source: Molecules. 2022 Oct 4;27(19):6566. doi: 10.3390/molecules27196566 (PMC9573107; doi:10.3390/molecules27196566)
Supplement: Supplementary file 1 [file molecules-27-06566-s001.zip › molecules-1939462-supplementary.pdf]

Supplementary Material

Multivariate Statistical Analysis for Mutual Dependence Assessment of Selected Polyphenols, Organic Acids and Metals in Cool-Climate Wines

Magdalena Fabjanowicz [1,*], Vasil Simeonov [2], Marcin Frankowski [3], Wojciech Wojnowski [1,4] and Justyna Płotka-Wasylka [5,*]

[1] Department of Analytical Chemistry, Faculty of Chemistry, Gdańsk University of Technology (GUT), 11/12 Narutowicza Street, 80-233 Gdańsk, Poland
[2] Faculty of Chemistry and Pharmacy, University of Sofia, 1 James Bourchier Blvd., 1126 Sofia, Bulgaria
[3] Deparment of Analytical and Environmental Chemistry, Faculty of Chemistry, Adam Mickiewicz University, Uniwersytetu Poznańskiego 8, 61-614 Poznań, Poland
[4] Department of Chemistry, University of Oslo, P.O. Box 1033-Blindern, 0315 Oslo, Norway
[5] Department of Analytical Chemistry, Faculty of Chemistry and BioTechMed Center, Gdańsk University of Technology (GUT), 11/12 Narutowicza Street, 80-233 Gdańsk, Poland
* Correspondence: magfabja@student.pg.edu.pl (M.F.); juswasyl@pg.edu.pl (J.P.-W.)

Table S1. Wine sample characteristics.

Label	Year	Type of wine	Voivodship	% Alcohol	Grape type	Sugar content
**1R**	2015	Red	Opolskie	11.0	Regent	dry
**2R**	2017	Red	Małopolskie	12.0	Regent	dry
**3R**	2014	Red	Lubelskie	12.1	Rondo	dry
**4R**	2013	Red	Małopolskie	12.5	Regent	dry
**5R**	2017	Red	Dolnośląskie	13.5	Dornfelder	dry
**6R**	2017	Red	Małopolskie	11.0	Rondo	dry
**7R**	2017	Red	Dolnośląskie	13.5	Pinot Noir	dry
**8R**	2016	Red	Zachodnio-pomorskie	13.0	Rondo/Regent	dry
**9R**	2015	Red	Opolskie	11.5	Rondo	dry
**10R**	2016	Red	Podkarpackie	12.5	Mix of 3 grapes	dry
**1W**	2016	White	Lubelskie	12.0	Johanniter	dry
**2W**	2017	White	Dolnośląskie	12.0	Riesling	semi-dry
**3W**	2016	White	Lubuskie	12.0	Pinot Gris, Riesling, Muscat Ottonel, Gewurztraminer	semi-dry
**4W**	2017	White	Małopolskie	12.0	Seyval Blanc, Hibernal, Johanniter, Solaris	semi-dry
**5W**	2016	White	Lubuskie	13.0	Pinot Gris	semi-dry
**6W**	2016	White	Lubelskie	12.5	Solaris	sweet
**7W**	2014	White	Małopolskie	12.0	Bianca	dry
**8W**	2017	White	Zachodnio-pomorskie	12.5	Solaris	dry
**9W**	2017	White	Podkarpackie	12.0	Mix of grapes	semi-sweet
**10W**	2015	White	Podkaprackie	11.5	Mix of 8 grapes	dry
**1Ro**	2014	Rosé	Małopolskie	10.5	Zweiglet	semi-dry
**2Ro**	2015	Rosé	Dolnośląskie	10.5	Regent	dry
**3Ro**	2016	Rosé	Podkarpackie	11.5	A mix of 3 grapes	dry

Table S2. Elemental composition of studied wine samples [µg/mL] and *[mg/mL].

Elements	1R	2R	3R	4R	5R	6R	7R	8R	9R	10R	1W	2W	3W	4W	5W	6W	7W	8W	9W	10W	1Ro	2Ro	3Ro
**Ag**	0.47	0.25	0.13	0.195	0.165	0.194	0.212	0.262	0.196	0.15	0.174	0.109	0.128	0.054	0.05	0.047	0.036	0.023	0.027	0.044	0.012	0.012	0.518
**Al**	367	156	79.7	108	653	244	539	136	494	235	695	1310	625	650	830	394	319	1230	334	379	556	764	521
**As**	80.7	26.3	15.6	10.4	11.5	5.98	7.25	4.08	6.42	6.44	3.92	5.94	13.1	3.66	9.16	3.64	3.6	4.42	6.42	4.03	2.6	7.89	4.36
**B**	3590	3510	5340	3910	4970	3310	7080	3890	7240	8380	3310	5170	7210	3440	7380	4990	1660	5740	4770	5320	3970	1750	7990
**Ba**	159	18	17.4	65	128	39.8	113	95.4	97.4	75.9	16.6	60.7	81.3	77.3	62.8	19.1	38.6	47.8	33.2	32.6	17.5	62.6	73.4
**Be**	0.279	0.132	0.059	0.213	0.733	0.249	0.63	0.656	0.189	0.081	3.33	1.95	2.07	4.2	2.82	1.42	1.08	10.1	0.917	0.778	0.754	7.6	0.763
**Ca**	46780	51310	51960	61260	56350	55070	66750	46720	51380	75170	93720	98460	87170	87440	72690	50850	64590	67780	71790	80050	48680	65150	78760
**Cd**	0.21	0.044	0.784	0.347	0.393	0.102	0.209	0.04	0.085	0.159	0.342	0.123	0.185	0.873	0.214	0.336	0.465	0.125	0.38	0.35	0.386	0.252	0.432
**Co**	2.53	0.446	0.624	1.32	3.03	0.992	3.17	0.965	2.07	1.14	2.35	3.56	3.74	3.15	5.82	2.77	3.52	2.23	2.01	2.74	3	1.88	1.69
**Cr**	5.84	4.53	8.83	6.69	9.14	7.41	12.8	5.78	6.03	11.3	15.2	17.9	14	7.58	8.51	11.1	2.78	4.55	14.7	16.4	6.38	21.6	6.95
**Cu**	<0.134	<0.134	<0.134	23.6	170	<0.134	53	33.1	46.9	73.2	<0.134	1290	133	<0.134	412	144	<0.134	15	743	260	<0.134	<0.134	<0.134
**Fe**	1920	562	556	480	370	786	436	346	2540	854	615	1160	4090	901	3550	576	877	732	1310	1780	994	565	890
**Hg**	0.126	0.117	0.098	0.087	0.08	0.063	0.059	0.046	0.05	0.059	0.06	0.04	0.047	0.05	0.035	0.024	0.02	0.02	0.02	0.024	0.015	0.012	0.055
**K ***	759.5	719.9	674.9	873.2	701.8	780.8	787.2	1038	664.3	962.3	655.4	940.7	838.4	753.5	853.2	506.6	547.5	474.6	672.3	701.6	558.6	551.3	648.1
**Li**	4.57	1.41	2.29	3.45	5.07	2.79	6.6	2.26	7.25	3.69	4.3	6.06	3.97	3.31	3.56	5.49	8.63	4.07	5.72	3.59	2.56	2.78	6.89
**Mg**	89300	82700	89000	95700	69700	83000	89900	94700	92700	101000	65800	80200	75100	83300	74900	93400	106000	94400	78400	79900	49700	87100	72800

**Mn**	1220	600	682	1340	1080	653	1870	1030	745	897	564	1960	794	1350	708	942	1630	1110	811	727	611	1420	1010
**Na**	1122	1142	232.2	737.2	12210	7256	6257	7632	1558	11790	2213	17840	17930	16990	10230	3856	256	11450	23580	22450	8347	15790	33990
**Ni**	67.1	17.8	31.2	299	28.6	24.5	39.2	25.6	54.2	40.3	39	41.2	21	31.8	41.7	53.3	147	27.3	71	68.5	29.2	23.5	39.4
**Pb**	6.71	1.16	9.11	3.42	3.14	1.77	1.87	2.84	5.19	1.78	4.45	2.48	8.41	3.48	8.41	2.98	4.81	2.89	5.08	7.07	1.29	3.09	6.59
**Sb**	0.838	0.691	0.593	0.69	0.56	0.547	0.481	0.486	0.582	0.558	0.574	0.64	0.612	0.569	0.579	0.549	0.572	0.515	0.722	0.668	0.559	0.566	0.753
**Se**	28.5	7.25	5.82	5.56	6.29	2.91	4.74	2.87	4.46	3.06	3.67	3.76	1.69	1.69	3.87	4.27	1.7	0.957	1.44	2.78	1.25	1.97	2.21
**Sn**	24.6	12.7	8.6	8.9	4.91	5.41	3.66	4.53	3.53	2.57	4.07	2.4	2.06	1.78	2.27	1.78	2.51	2.62	5.12	6.81	1.37	4.34	4.72
**Sr**	481	175	637	244	372	302	387	329	325	298	284	348	164	253	157	281	337	206	239	207	116	376	251
**Ti**	20.3	13.5	5.68	8.11	9.31	16.4	5.13	8.48	105	20.2	13.8	18.9	52.5	13.7	25.8	19.8	7.48	9.51	48.4	25.6	9.37	10.5	28.9
**Tl**	1.62	0.826	0.373	0.622	0.362	0.791	0.477	0.294	0.467	0.735	0.251	0.54	0.412	1.04	0.592	0.278	1.11	0.18	0.276	0.359	0.855	0.681	0.254
**V**	3.08	4.51	0.51	3.14	4.13	19.6	2.05	0.583	4.36	7.19	6.85	9.78	72.2	6.21	10.3	5.31	0.973	2.48	8.92	2.88	1.32	25.8	1.52
**Zn**	721	238	544	1470	1570	842	1440	632	916	544	899	928	733	1210	566	1020	1670	940	547	673	591	876	951
**Zr**	12.7	5.04	3	2.52	3.96	3.3	3.53	2.43	3.82	2.95	11.8	8.29	8.19	9.19	11	4.31	2.03	9.17	12.1	10.3	9.85	7.01	12.2

Table S3. Organic acids and polyphenols concentration in studies wine samples [mg/mL].

Sample	Lactic acid	Succinic acid	Fumaric acid	Malic Acid	Tartaric acid	Citric acid	protocatechuic	p-coumaric acid	gallic acid	Ferulic acid	Caffeic acid	Sinapic acid	Resveratrol	Catechin
**1R**	260.66	259.29	0.14	23.46	58.89	4.1	1.83	7.82	2.36	0.175	25.18	0.89	2.88	454.14
**2R**	340.35	457.53	0.14	117.17	46.77	4.1	2.5	10.62	2.21	0.175	15.41	0.91	2.46	336.41
**3R**	299.48	456.33	0.14	54.2	44.75	26.82	5.22	2.99	2.49	0.175	9.14	0.9	2.96	383.03
**4R**	315.55	465.43	0.14	570.9	48.47	54.29	6.75	11.65	4.97	0.175	21.03	0.96	2.98	964.97
**5R**	305.59	466.05	0.14	37.89	65.03	23.49	2.48	4.39	2.51	0.175	12.13	0.92	2.33	66.86
**6R**	316	387.63	0.14	31.06	39.31	4.1	0.18	12.33	2.72	0.175	25.25	0.86	2.54	140.49

**7R**	356.18	350.78	0.14	115.21	39.85	4.1	1.02	7.15	6.59	0.175	30.74	0.89	5.09	6225.86
**8R**	333.27	355.33	0.14	217.65	41.47	32.92	5.26	10.87	6.11	0.175	8.36	0.96	4.02	2860.05
**9R**	309.74	401.66	0.14	20.94	46.76	4.1	2.24	7.12	1.02	0.175	14.27	0.85	2.28	10.35
**10R**	439.32	370.66	0.14	23.25	43.3	4.1	0.18	12.44	0.86	0.175	10.2	0.95	4.7	5524.8
**1W**	45.2	256.4	0.14	870.31	75.91	162.73	0.18	1.11	0.24	0.175	8.88	0.84	2.27	119.24
**2W**	328.47	316.16	0.14	175.42	33.2	4.1	0.18	0.8	0.19	0.175	8.31	0.88	2.25	41.96
**3W**	116.99	310.94	2.06	901.71	34.16	206.2	0.18	0.94	0.32	0.175	6.17	0.86	2.27	16.78
**4W**	53.88	271.66	1.55	1080.7	40.47	222.18	0.18	2.23	0.47	0.175	7.57	0.89	2.38	34.95
**5W**	79.63	474.23	3.1	1420.78	35.16	329.34	0.18	0.91	0.61	0.175	7.69	0.86	2.36	29.62
**6W**	60.53	252.6	0.14	921.27	42.6	172.61	0.18	0.14	0.12	0.175	5.78	0.89	2.24	34.19
**7W**	98.75	576.36	1.47	1158.28	47.86	311.8	1.34	1.23	0.21	0.175	6.33	0.87	2.51	110.38
**8W**	75.89	225.71	0.14	1027.19	33.08	172	0.18	0.14	0.09	0.175	4.45	0.84	2.2	1
**9W**	225.58	338.01	2.77	1661.21	37.46	296.92	0.18	1.12	0.25	0.175	5.27	0.91	2.25	14.88
**10W**	315.29	385.46	1.67	1640.32	34.48	321.55	0.18	2.22	0.3	0.175	6.22	0.86	2.31	29.89
**1Ro**	125.62	338.06	0.14	867.11	44.23	163.33	0.18	0.78	0.1	0.175	6.52	0.84	2.32	1
**2Ro**	221.14	949.65	1.38	2185.42	78.88	370.65	2.37	22.34	1.18	0.175	13.58	1.03	2.29	38.21
**3Ro**	319.83	248.07	0.14	205.7	35.53	187.59	0.18	0.14	0.12	0.175	5.49	0.9	2.21	1

Table S4. Input table.

LA	SA	FA	MA	TA	CA	protocat	p-CoumA	GA		
CafA	SinA	Res	CAT	Ag	Al	As	B	Ba	Be	
Ca	Cd	Co	Cr	Cu	Fe	Hg	K	Li	Mg	Mn
Na	Ni	Pb	Sb	Se	Sn	Sr	Ti	Tl	V	Zn	Zr

Table S5. Factor loadings.

Factor Loadings (Varimax normalized) Extraction: Principal components (Marked loadings are >.700000)				
	**Factor 3**	**Factor 4**	**Factor 1**	**Factor 2**
**LA**	0.137	0.152	**0.731**	-0.197
**SA**	-0.052	**0.715**	-0.280	-0.008
**FA**	-0.010	-0.048	-0.432	0.598
**MA**	-0.230	0.204	**-0.748**	0.416
**TA**	0.252	0.542	-0.306	-0.211
**CA**	-0.195	0.066	**-0.745**	0.459
**protocat**	0.175	0.451	0.199	-0.518
**p-CoumA**	0.107	**0.701**	0.214	-0.280
**GA**	0.074	0.446	**0.711**	-0.352
**CafA**	0.407	0.403	0.491	-0.212
**SinA**	0.019	**0.721**	0.141	-0.095
**Res**	-0.120	0.315	**0.765**	-0.142
**CAT**	-0.216	0.271	**0.764**	-0.039
**Ag**	0.602	-0.143	0.493	-0.192
**Al**	-0.195	-0.075	-0.181	0.641

**As**	0.639	0.050	0.078	-0.101
**B**	-0.131	-0.532	0.543	0.329
**Ba**	0.490	0.245	0.533	0.290
**Be**	-0.273	0.222	-0.481	0.288
**Ca**	-0.209	-0.141	-0.063	**0.711**
**Cd**	-0.008	0.050	-0.401	-0.053
**Co**	-0.006	-0.113	-0.207	**0.725**
**Cr**	-0.164	0.230	-0.093	0.549
**Cu**	-0.072	-0.135	0.106	0.598
**Fe**	0.286	-0.448	-0.074	0.556
**Hg**	0.603	-0.003	0.378	-0.448
**K**	0.105	0.028	**0.729**	0.157
**Li**	-0.007	-0.001	0.160	0.449
**Mg**	-0.010	0.467	0.313	-0.113
**Mn**	-0.012	0.631	0.285	0.434
**Na**	-0.169	-0.248	-0.058	0.595
**Ni**	0.143	0.356	-0.002	-0.074
**Pb**	0.408	-0.309	-0.219	0.313
**Sb**	**0.778**	-0.196	-0.111	0.108
**Se**	**0.915**	0.084	0.161	-0.131
**Sn**	**0.900**	0.098	0.074	-0.300
**Sr**	0.326	0.412	0.252	-0.205
**Ti**	0.116	-0.433	0.088	0.277
**Tl**	0.610	0.277	-0.075	-0.083
**V**	0.020	-0.079	-0.091	0.427

**Zn**	-0.131	0.491	0.075	0.171
**Zr**	0.339	-0.431	-0.428	0.467
**Expl.Var %**	18.8	17.2	26.3	23.7

Table S6. ReliefF scoring of chemical elements with relation to the concentration of resveratrol.

ReliefF	Feature
0.159303	Zr
0.157549	Hg
0.147362	Ag
0.139947	Ca
0.098372	Be
0.074911	Na
0.0644	Co
0.05065	Al
0.04849	Sr
0.040874	Cr
0.037904	Sn
0.035769	Se
0.031664	Ba
0.018398	Mg
0.015779	K
0.015433	As
0.012155	Fe
0.009751	Cu
0.009501	V
0.00694	Pb
0.005058	Li
0.002427	Ti
-0.002202	B
-0.006083	Cd
-0.006937	Sb
-0.01024	Ni
-0.011059	Tl
-0.02038	Zn
-0.025068	Mn

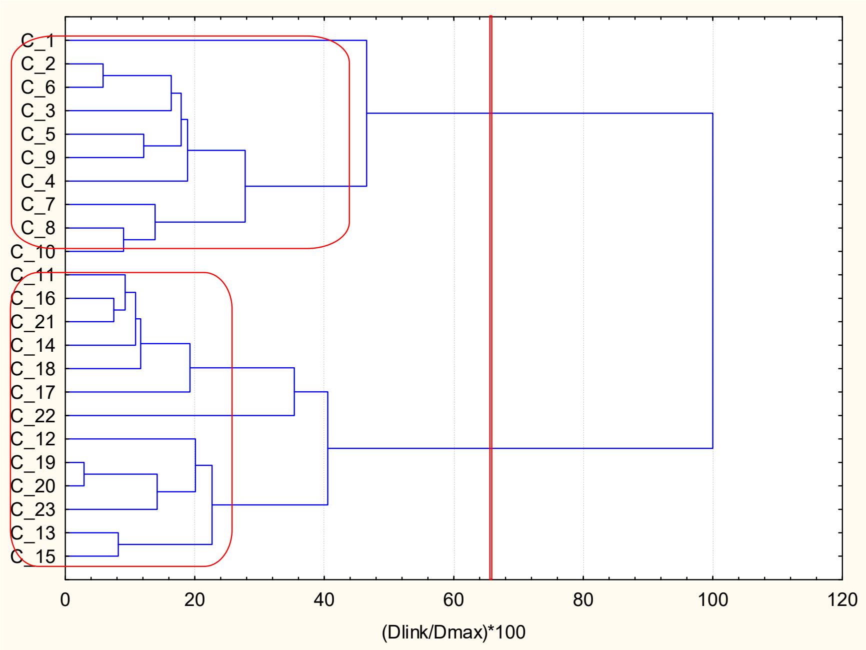

Figure S1. Hierarchical dendrogram for clustering of 23 wine samples (objects).

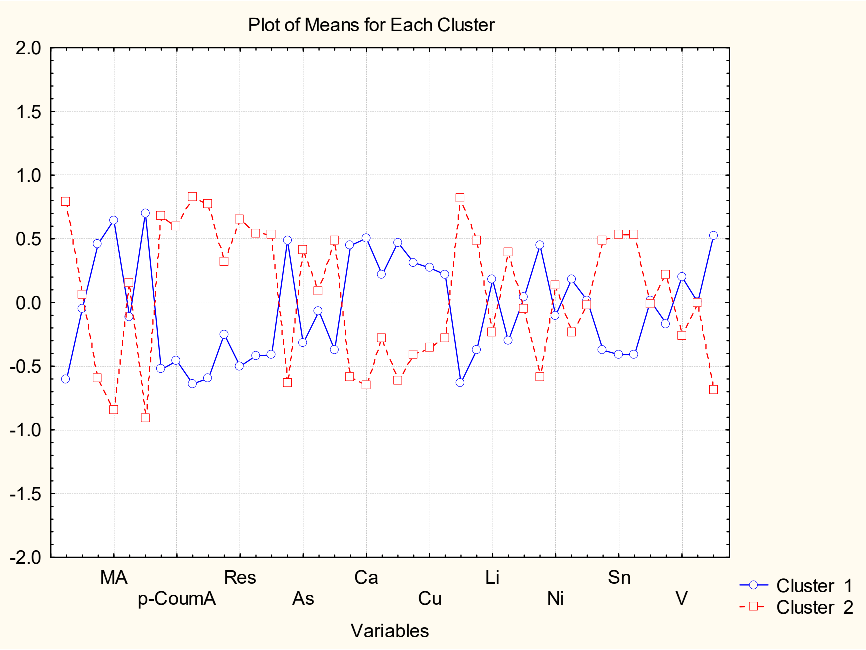

Figure S2. Plot of mean values (standardized) for each variable for each identified cluster.

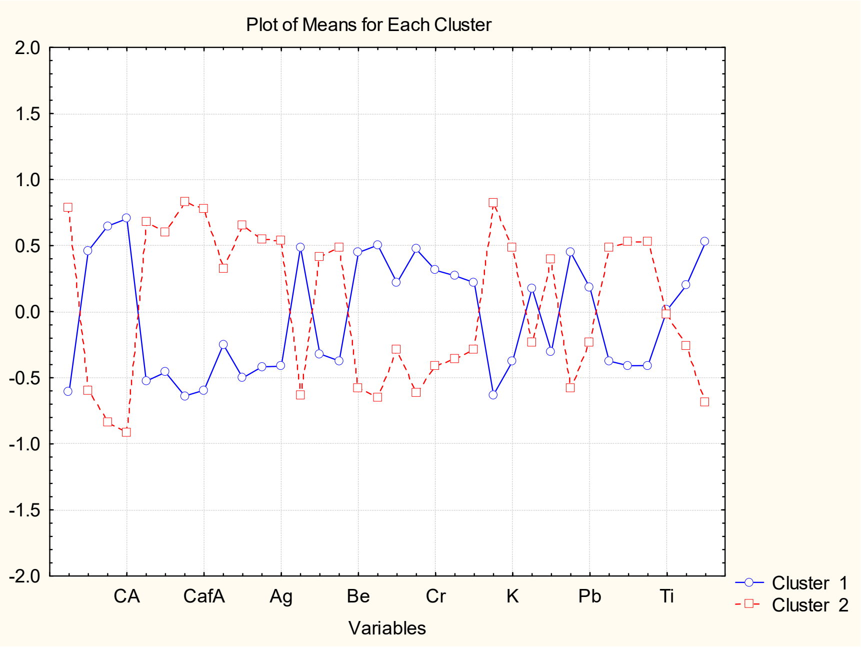

Figure S3. Plot of mean values (standardized) for each variable (reduced number) for each identified cluster.
